# Sensing surface mechanical deformation using active probes driven by motor proteins

**DOI:** 10.1038/ncomms12557

**Published:** 2016-10-03

**Authors:** Daisuke Inoue, Takahiro Nitta, Arif Md. Rashedul Kabir, Kazuki Sada, Jian Ping Gong, Akihiko Konagaya, Akira Kakugo

**Affiliations:** 1 Faculty of Science, Hokkaido University, Sapporo 060-0810, Japan; 2 Applied Physics Course, Gifu University, Gifu 501-1193, Japan; 3 Graduate School of Chemical Sciences and Engineering, Hokkaido University, Sapporo 060-0810, Japan; 4 Faculty of Advanced Life Science, Hokkaido University, Sapporo 060-0810, Japan; 5 Department of Computational Intelligence and Systems Science, Tokyo Institute of Technology, Yokohama 226-8501, Japan

## Abstract

Studying mechanical deformation at the surface of soft materials has been challenging due to the difficulty in separating surface deformation from the bulk elasticity of the materials. Here, we introduce a new approach for studying the surface mechanical deformation of a soft material by utilizing a large number of self-propelled microprobes driven by motor proteins on the surface of the material. Information about the surface mechanical deformation of the soft material is obtained through changes in mobility of the microprobes wandering across the surface of the soft material. The active microprobes respond to mechanical deformation of the surface and readily change their velocity and direction depending on the extent and mode of surface deformation. This highly parallel and reliable method of sensing mechanical deformation at the surface of soft materials is expected to find applications that explore surface mechanics of soft materials and consequently would greatly benefit the surface science.

## Abstract (web summary)

It is challenging to characterize the mechanical properties of soft surfaces owing to the coupling between surface deformation and elasticity of bulk materials. Here, Inoue *et al*. use motile cytoskeletal filaments as active probes, whose direction reflects the stress field experienced by the surface.

## Graphical abstract

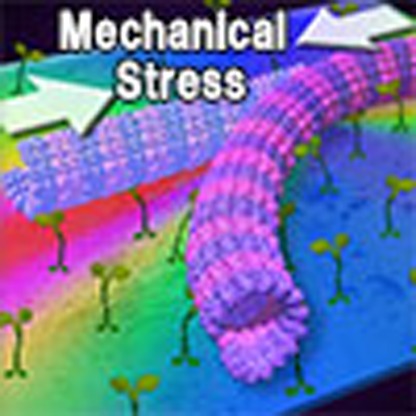

The self-propelled micromotors offer great potential for diverse practical applications, including targeted drug delivery, bio-imaging and monitoring of surroundings^1^,^2^,^3^,^4^,^5^,^6^,^7^,^8^. Probing surrounding environment using a large number of active probes provides robust sensing and monitoring, as well as high spatial resolution and acquisition rate. Among the self-propelled micromotors, motor protein systems, for example, actin/myosin^9^, microtubule/kinesin^10^ and microtubule/dynein^11^, offer unique features which are particularly suitable for sensing and monitoring^11^,^12^,^13^,^14^,^15^,^16^. In an *in vitro* gliding assay, cytoskeletal filaments (for example, microtubules or actin filaments) are propelled by their associated motor proteins (for example, kinesin, dynein or myosin), which are adhered to a substrate. During the propulsion, the free end of the leading tip of a gliding cytoskeletal filament undergo thermal fluctuations until it binds to a motor protein; the remaining part of the filament is anchored to the other motor proteins^17^. This anchored ‘search-and-catch' mechanism provides a potential means for sensing the local environment of a submicrometer region. Thus, motor protein systems have been employed as active probes to examine the topographical features and chemical properties of a surface^12^,^13^,^14^,^15^,^16^.

Here, we pursue the potential of utilizing gliding cytoskeletal filaments as active probes, and extend the concept for sensing mechanical stress at the surface of a soft material. Fluorescently labelled microtubules are propelled by kinesins adhered to the surface of a stretchable substrate. The response of the gliding microtubules to mechanical deformation of the substrate is monitored using fluorescence microscopy. The gliding velocity of the microtubules is found tightly correlated to the extension or compression of the substrate. This finding enables measurements of the extension or compression of the substrate through measurements of gliding velocity of the microtubules. The randomly wandering microtubules become oriented depending on the direction of deformation of the substrate, enabling us to determine the direction of the stress field experienced by the substrate. This work introduces a new concept for characterizing surface mechanical deformation of soft materials by employing motor protein-driven cytoskeletal filaments, and at the same time offers valuable insights in the dynamic behaviour of motor protein systems under mechanical stress.

## Results

### *In vitro* gliding assay on a stretchable substrate

**Figure 1:**
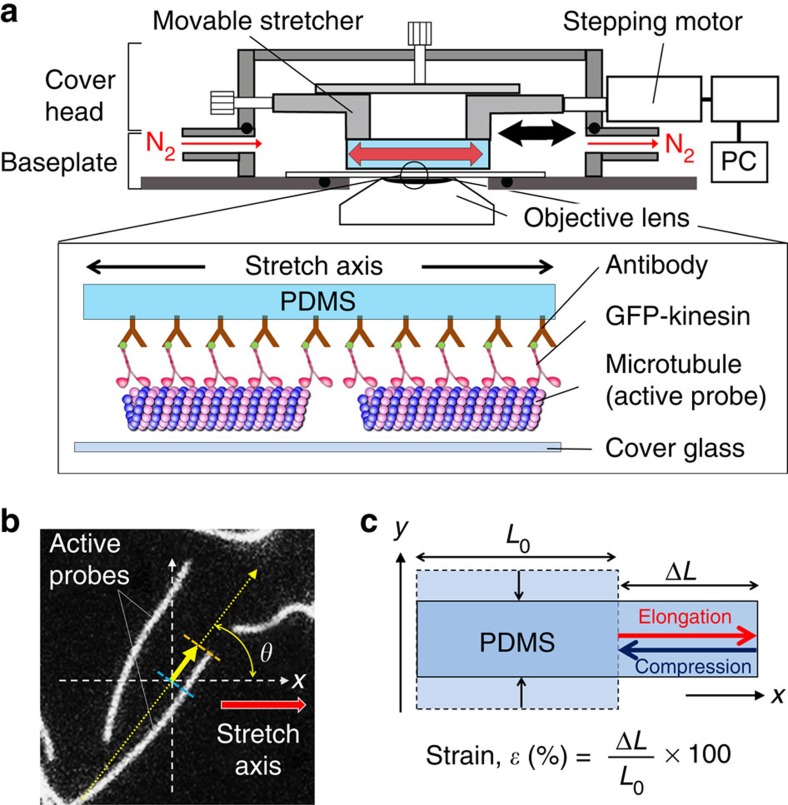
Elongation and compression of the gliding assay substrate. (**a**) Schematic diagram showing the design of the stretch chamber and the *in vitro* gliding assay system of active probes (microtubules) on an elastic substrate polydimethylsiloxane (PDMS). (**b**) Representative fluorescence microscopy image of gliding active probes. Tensile or compressive strain was applied to the PDMS substrate along the *x* axis. *θ* is the angle between the track of the front end of the active probe (yellow arrow) and the stretch axis (red arrow) (range of *θ* is from 0° to 360°); this angle was measured in 10 s intervals. The orange dotted line represents the front position of the microtubule, and the blue dotted line represents the position 10 s before. (**c**) Definition of stretching strain, *ɛ*, where *L*_0_ is the initial length of the gliding assay substrate, and Δ*L* is the change in length of the substrate after application of strain.

The *in vitro* gliding assay of microtubules was performed on polydimethylsiloxane (PDMS) which is a soft and stretchable elastomeric material. A stretch chamber equipped with a computer-controlled stepping motor was developed to stretch the substrate of the *in vitro* gliding assay (Fig. 1a; Supplementary Fig. 1)^18^,^19^. A sheet of PDMS was fixed horizontally at the movable stretcher of the stretch chamber (Fig. 1a; Supplementary Fig. 2a). Anti-GFP antibodies were deposited on the PDMS surface; then, green fluorescent protein fused kinesin-1 (GFP-kinesin) motor proteins were applied to the PDMS. Next, paclitaxel-stabilized fluorescent dye-labelled microtubules were deposited on the kinesin-coated PDMS surface, and these microtubules worked as ‘active probes' (Fig. 1a; Supplementary Fig. 2a). After the sample preparation, the stretch chamber was closed, and humid nitrogen gas was kept passing through the chamber to remove oxygen from the chamber, which allowed performing the *in vitro* gliding assay of the active probes on the PDMS surface in an inert atmosphere. The inert atmosphere prevents oxidative damage of microtubules and kinesins and thereby can significantly prolong the lifetime of the gliding assay^20^ (Supplementary Note 1; Supplementary Movie 1). Finally, the gliding assay of the active probes was initiated by adding adenosine triphosphate (ATP) to the assay system (Supplementary Fig. 2b).

### Effect of substrate deformation on the velocity of probes

**Figure 2:**
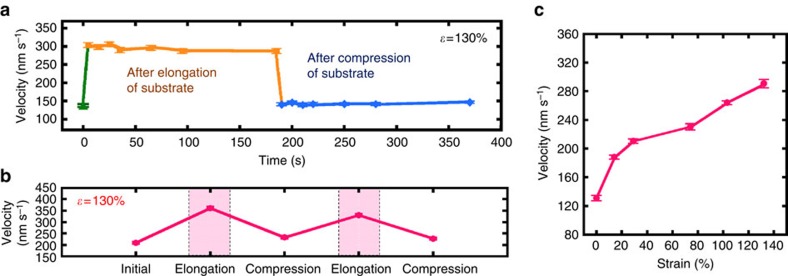
Change of the velocity of probes upon substrate deformation. (**a**) Time course of the active probe velocity after elongation and compression of the gliding assay substrate. (**b**) Reversible changes in the active probe velocity after the repeated application of tensile and compressive stress. The gliding assay substrate was elongated and compressed at a strain rate of 0.6% s^−1^. (**c**) Standard curve showing the relationship between the stretching strain and the velocity of active probes. Error bar: s.e.

Upon addition of the ATP (at 0 min), the active probes started gliding on the kinesin-coated PDMS surface at a constant velocity (mean velocity of 140±4.0 nm s^−1^ at 25 °C) without showing any specific directional preference (Fig. 1b). First, we describe the effect of elongation and compression of the gliding assay substrate on the velocity of active probes. The PDMS substrate was elongated with the strain of *ɛ*=130% with respect to its initial length at a strain rate of 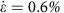 S^−1^; then, the substrate was compressed back to its initial length at the same strain rate (note: in this work the term ‘strain' indicates the strain applied to the PDMS substrate (Fig. 1c)). The velocity of the active probes increased by the elongation of the substrate and decreased by the compression of the substrate (Fig. 2a). The highest velocity of the active probes (300±6.0 nm s^−1^) was observed at the strain of *ɛ*=130%, and this value was larger than twice the initial velocity (140±4.0 nm s^−1^) observed in the absence of applied strain. The velocity of the active probes decreased by the compression of the substrate, finally reached to the initial velocity observed in the absence of applied strain to the substrate and remained unchanged over time. Thus changing the mode of deformation of the substrate it is possible to reversibly regulate the velocity of the active probes. We also investigated the repeatability of the reversible regulation of velocity of the probes by applying tension and subsequent compression at the substrate in a cyclic manner, and found that the velocity of the active probes can be regulated repeatedly by using the stretch chamber (Fig. 2b).

To understand the origin of the velocity change of active probes upon deformation of the gliding assay substrate, we monitored the fluorescence intensity of GFP-kinesins on the substrate during this process. We found that as the tensile strain at the substrate increased, the fluorescence intensity of GFP-kinesins gradually decreased, whereas the velocity of the active probes increased (Fig. 2c). Since the fluorescence intensity depends on the kinesin density on the substrate surface, at this stage it seems that the change in velocity of the active probes is correlated to the kinesin density on the substrate surface. To confirm the relationship between the velocity of the active probes and the kinesin surface density, we performed *in vitro* gliding assays on PDMS substrate by varying the concentration of GFP-kinesin solution fed into the gliding assay system and measured the velocity of the active probes and fluorescence intensity of GFP-kinesins for each case. By using quartz crystal microbalance (QCM), we estimated kinesin density on the PDMS surface for each of the kinesin concentrations fed into the gliding assay system^18^,^19^, which was finally correlated to the measured fluorescent intensity of GFP-kinesins (Supplementary Fig. 3a). From this estimation, we prepared a standard curve that correlated the velocity of the active probes with the GFP-kinesin surface density (Supplementary Fig. 3b). The velocity of the active probes was strongly dependent on the kinesin density on PDMS surface and increased sharply when the kinesin density was increased from 70 to 112 μm^−2^. The highest velocity of the active probes (410±4.0 nm s^−1^) was observed at a kinesin density of 112 μm^−2^; the velocity gradually decreased as the kinesin density was increased further. This velocity was higher than twice the velocity of the active probes (190±3.0 nm s^−1^) obtained at the highest kinesin density employed in this work (560 μm^−2^). This change in the active probe velocity in response to the change in kinesin density corresponds well with the results of previous reports^21^,^22^. Finally, using the standard curve shown in Supplementary Fig. 3a and the values of the fluorescence intensity of GFP-kinesins at different strains at the gliding assay substrate, we estimated the kinesin density on the substrate surface at different strains (Supplementary Fig. 3c). The change in the active probe velocity under applied stress correlated well with the change in the kinesin density on the gliding assay substrate. It should be mentioned that, the change in surface hydrophobicity, which may affect the protein mobility^23^,^24^, was not observed upon stretching of PDMS^25^. Therefore, the change in velocity of active probes on elongation and compression of the gliding assay substrate is due to the change in the density of kinesin on the substrate. In addition, since smoothness of the movement of active probes is a factor for the precise measurement, we also evaluated the smoothness of the movement of active probes in terms of motional diffusion and found that there was no considerable difference in the smoothness of movement of active probes after elongation and compression of the gliding assay substrate (see Supplementary Note 2; Supplementary Figs 4 and 5).

### Effect of substrate deformation on the direction of probes

**Figure 3:**
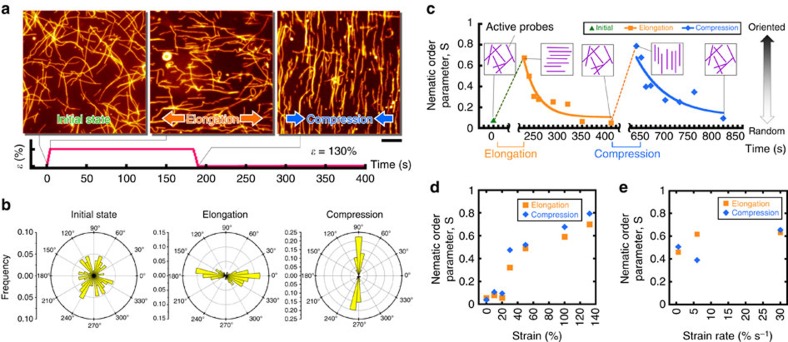
Change of the direction of probes upon substrate deformation. (**a**) Fluorescence microscopy images showing orientation of active probes at different substrate conditions. The active probes moved randomly on the gliding assay substrate before the substrate deformation (left). The active probes were oriented parallel (middle) and perpendicular (right) to the direction of substrate elongation and compression, respectively. For both the elongation and compression, 130% strain was applied to the substrate at the strain rate of 0.6% s^−1^. Scale bar, 10 μm. (**b**) Circular histograms of orientation angle of the active probes corresponding to the fluorescence microscopy images shown in **a**. (**c**) Time course of the nematic order parameter, *S*, of the active probes after elongation and compression of gliding assay substrate. The *S* was calculated from the orientation angle, *α* (0°≤*α*≤180°), of the active probes at various time points after the elongation and compression of the substrate (strain: 130%). (**d**) Effect of the elongation and compression of the gliding assay substrate on the *S* of the active probes. These experiments were performed at a fixed strain rate of 0.6% s^−1^ and in every case the *S* was measured just after stopping the elongation or compression of the substrate. (**e**) Effect of strain rate on the *S* of the active probes. For a fixed strain of 130%, no considerable effect was observed when the strain rate was varied from 0.6 to 30.0% s^−1^. For different strain rates the *S* was measured just after stopping the elongation or compression of the substrate.

We investigated the effect of elongation and compression of the gliding assay substrate on the direction of movement of the active probes. Initially, in the absence of any applied stress, the active probes moved randomly without showing any preferred direction (Fig. 3a, left). When the substrate was elongated or compressed, the direction of movement of the active probes changed. At a strain of *ɛ*=130%, the active probes were aligned parallel to the direction of stretching axis during substrate elongation and perpendicular to the stretching axis during compression (Fig. 3a middle, right; Supplementary Movies 2 and 3). To evaluate the orientation behaviour of the active probes, we measured the orientation angle of the active probes, *θ* (range of *θ* is from 0° to 360°; Fig. 1b), which is the angle between the track of the leading end of active probes and the stretch axis within 10 s, and we plotted the orientation angle on a circular histogram (Fig. 3b). Initially, the orientation angle of the active probes was dispersed around the circular histogram (Fig. 3b, left), but the active probes adopted an angle corresponding to the parallel or perpendicular orientation relative to the stretch axis after the substrate was elongated or compressed, respectively (Fig. 3b middle, right). Here, we evaluated the degree of orientation of the active probes by measuring the nematic order parameter, *S* which is defined as


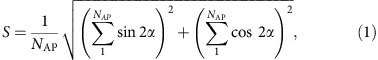


where *N*_AP_ is the total number of active probes, and *α* is an angle that ranges from 0° to 180° depending on the symmetry of the distribution of the active probe angle with respect to the horizontal axis^26^,^27^. The *S*=0 or 1 represents the random or oriented alignment of the active probes, respectively. Here, to analyse the direction of motion of the active probes, we evaluated the change in the *S* with time after the substrate was no longer deformed. For both elongation and compression of the substrate, the *S* increased to a value close to 1 immediately after the substrate was deformed. Then, the *S* value returned to almost 0 within a few minutes (Fig. 3c). The decrease in the *S* implies that the ordered alignment of the active probes was disrupted into a random state within a short time. We evaluated the characteristic relaxation time, *τ* that is the time by which the orientation of active probes became 1/*e* (37%) of the total change in the *S*. For elongation and compression of the substrate, *τ* were calculated as *τ*_elongation_=25.6 s and *τ*_compression_=48.9 s, respectively. A difference in the relaxation time for elongation and compression is observed where the major effect is due to the difference in velocity and fluctuation or detachment of the probes, while other factors such as rigidity of the probes were found negligible (Supplementary Note 2; Supplementary Figs 6–8). From the alignment of the active probes along the stretching axis, as discussed above, one may suppose that the probes prefer areas with lower kinesin density. To understand whether kinesin density has any influence on the alignment of the probes, or in other words, the probes preferentially move depending on gradient in kinesin density, we took into consideration the number of kinesins bound to a single probe after stretching the substrate (130% strain). The difference in the number of kinesin bound to a single probe aligned along the parallel and perpendicular direction to the stretching axis is found quite negligible. Moreover, no significant difference was observed in the velocity of the probes at different directions before and after stretching of the gliding assay substrate. Thus the preferential alignment of the probes upon stretching or compression of gliding assay substrate seems not to be related to the change in kinesin density or heterogeneity of kinesin density (Supplementary Note 2; Supplementary Figs 9 and 10).

Next, we evaluated the effect of stretching strain and strain rate on the alignment of the active probes in terms of the *S*. We found that randomly moving active probes started to align as the stretching or compressive strain was increased (Fig. 3d). When the stretching or compressive was increased to *ɛ*=130%, most of the active probes assumed a parallel or perpendicular orientation respectively relative to the stress axis (Fig. 3d). For a fixed strain of *ɛ*=130%, we then investigated the effect of the stretching and compression rate on the orientation of the active probes by varying the strain rate over one order of magnitude, for example, from 0.6 to 30.0% s^−1^. The strain rate had no effect on the *S* within the range studied, where the *S* was measured just after stopping elongation or compression of the substrate (Fig. 3e). Thus, it is the strain, not the strain rate, which played the key role in the orientation of the active probes upon elongation or compression of the gliding assay substrate.

### Buckling of non-motile probes upon substrate compression

**Figure 4:**
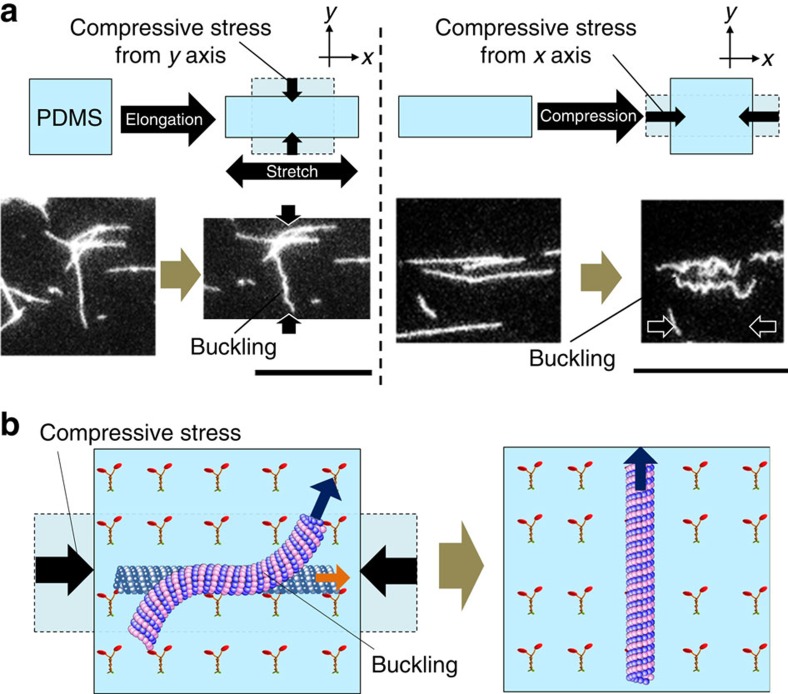
Buckling of non-motile probes upon compression of substrate. (**a**) Fluorescence microscopy images of buckled probes which were immobilized to the substrate due to the absence of ATP in the gliding assay system. During elongation of the substrate along the *x* axis, the probes which were aligned perpendicular to the stretch axis underwent buckling due to compression of the substrate along the *y* axis (left). By contrast, during compression of the substrate along the *x* axis, the probes that were parallel to the stretch axis underwent buckling by the compressive stress along the *x* axis (right). Scale bar, 20 μm. (**b**) Schematic illustration describing the mechanism of change of the direction of movement of buckled active probes on compression of the substrate.

To elucidate the orientation mechanism of the active probes under deformation of the gliding assay substrate we performed a control experiment where the probes were immobilized to the substrate in the absence of ATP, and tensile and compressive stress were applied to the substrate at a strain of *ɛ*=130%. When the substrate was elongated (*x* axis was the stretch axis), the non-motile probes which had been directed towards the *y* axis of the substrate, formed a buckled structure (Fig. 4a). But when the substrate was compressed (*x* axis was the compression axis), the probes that had been directed toward the *x* axis showed buckled structure (Fig. 4a). The buckling of the non-motile probes could be accounted for in the light of our recent report where we revealed how compressive stress at the substrate resulted in buckling of the non-motile probes^19^. The buckling of the non-motile probes observed in the present work is the result of compressive stress at the probes that originated from contraction of the substrate; the probes underwent buckling whenever they were subjected to compressive stress due to compression of the substrate irrespective of the mode of deformation of the substrate by the applied stress (Fig. 4). The probes are sensitive to compressive stress along their longitudinal axis because the probes are one dimensional (aspect ratio∼10^3^). As reported in our recent work, compressive stress induced buckling of the probes is accompanied by a simultaneous accumulation of bending energy^19^. It could be assumed that in the presence of ATP, the moving active probes also underwent the compressive stress induced buckling and changed their moving direction, thereby minimizing the accumulated bending energy (Fig. 4b). However, it was difficult to experimentally confirm the effect of bending on the orientation or change in moving direction of the active probes because of the difficulty in monitoring the active probes during elongation and compression of the gliding assay substrate.

### Orientation of simulated probes upon substrate compression

**Figure 5:**
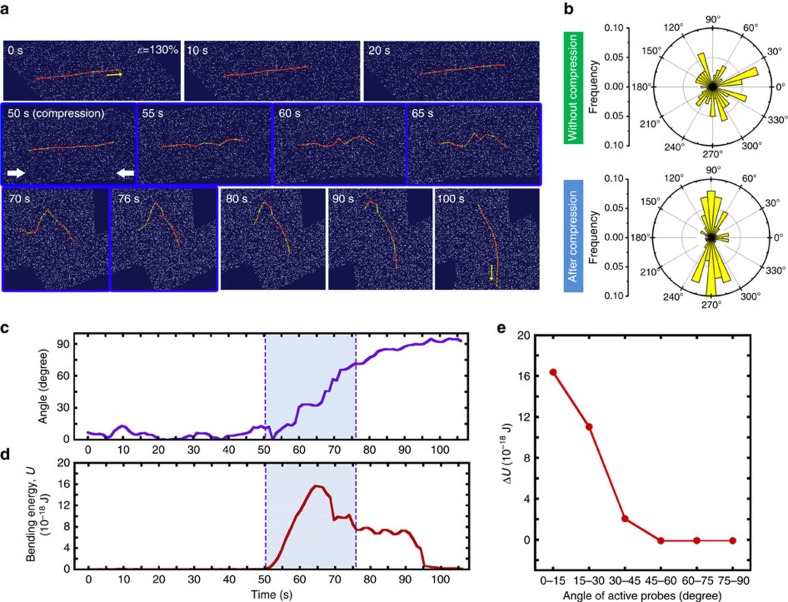
Effect of compression of substrate on the simulated probes. (**a**) Time series showing the conformations of a representative simulated active probe. The red line represents a simulated active probe gliding on a kinesin-coated surface (white dots). Green dots indicate kinesin binding to the active probe. Yellow arrows represent the movement direction of the simulated active probe. (**b**) Circular histogram representing the orientation angle distribution of the simulated active probes before and after compression of the substrate. (**c**) Time evolution of the angle and (**d**) the bending energy, *U*, of a representative simulated active probe shown in **a**. Compressive stress was continuously applied to the active probe from 50 to 76 s indicated by the shaded regions in **c** and **d**. (**e**) Difference in the bending energy, Δ*U*, of simulated active probes moving in different directions. The angle of the active probes at 50 s were binned into six groups as 0-15°, 15–30°, 30–45°, 45–60°, 60–75° and 75–90°.

To test the hypothesis that the active probes are oriented to the direction which minimized their accumulated bending energy, we performed a simulation study. We adopted and modified a computer simulation of an *in vitro* gliding assay of microtubules for this study (see ‘Methods' and Supplementary Figs 11 and 12)^28^. We simulated the alignment of the active probes induced by the substrate compression. The simulated active probes were directed perpendicular to the compression axis of the substrate during the compression, showing an agreement to the experimental observation discussed above (Supplementary Movie 4). The simulation enabled us to investigate the detailed mechanism of the alignment change of the active probes due to the deformation of the substrate. The time evolution of the conformations of a representative simulated active probe during substrate compression was investigated, where the probe was moving initially against the direction of compression (Fig. 5a). The computer simulation revealed that upon compression of the substrate, the simulated active probe that was initially moving along the compression axis buckled, and eventually its direction of movement became perpendicular to the compression axis (Fig. 5b,c). We also calculated the *S* for the simulated active probe during substrate compression and found a trend that is similar to the experimental results already described above (Supplementary Fig. 13). The simulation also revealed that during this change in direction of movement, there was a marked difference in the time evolution of the angle and bending energy, *U*, of the simulated active probe because the probe was bent when the substrate was compressed (Fig. 5c,d). Given this finding, we next considered a change in the bending energy of hundreds of active probes that were moving randomly just before the substrate compression. The difference in bending energy of the active probes before and after the substrate compression, Δ*U*, was smaller for active probes that were moving perpendicular to the compression axis at the onset of the compression than for the active probes that were moving parallel to the axis (Fig. 5e). This result clearly reveals that upon deformation of the substrate the active probes aligned themselves along the direction that minimized their accumulated bending energy.

Moreover, based on the buckling of probes under compressive stress and the outcome of simulation study discussed above, the strain rate independence of the nematic order parameter, *S* can be explained as follows. As already discussed, there was no considerable effect of strain rate on the *S* of the active probes which implies that alignment of the active probes through release of bending energy was slower, or in other words, the active probes did not have enough time to escape from the buckled state. Here, we assess the minimal strain rate which could affect the *S*. As reported in literature, a kinesin moves along a microtubule over a so called run length of ∼500 nm and detach from the microtubule^29^. Taking into account a translational speed of kinesins of 200 nm s^−1^ (as described above), a kinesin motor remains attached to a microtubule for ∼2.5 s. On the other hand, our recent work^19^ showed that the critical buckling strain, that is, the minimum strain required to buckle a microtubule, is ∼1%. Thus it is clear that in order to buckle a microtubule, the ∼1% strain has to reach within ∼2.5 s. Therefore, the minimum strain rate is estimated to be ∼0.4% s^−1^. This estimated strain rate is smaller than the lowest strain rate investigated in our work (0.6% s^−1^). At present confirmation of the actual minimal strain rate through experimentation is limited by the lowest strain rate that could be applied using the stepping motor used in this work (0.6% s^−1^).

### Demonstration of sensing surface mechanical deformation

**Figure 6:**
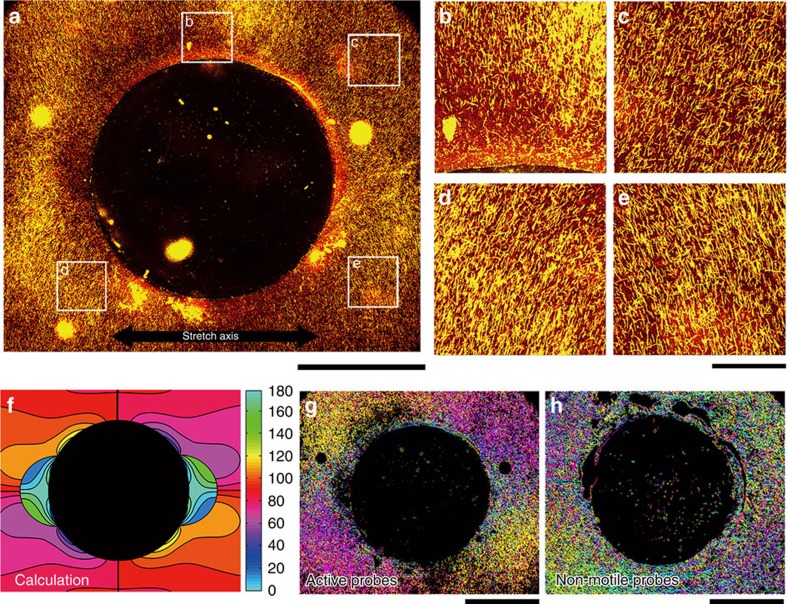
Orientation of the probes in an inhomogeneous stress field. (**a**) Fluorescence microscopy images showing the orientation of active probes, with respect to the stretch axis indicated by the black arrow, in an inhomogeneous stress field. Scale bar, 500 μm. (**b**–**e**) The enlarged views of the areas marked by white squares in **a** show the orientation of the probes in those areas. Here **b**–**e** represent the top left, top right, bottom left and bottom right area respectively in **a** marked by the white squares. Scale bar, 100 μm. Here the gliding assay substrate with a hole was elongated with the strain of 75% at the strain rate of 0.6% s^−1^, which was followed by compression of the substrate to its initial length at the same rate. The fluorescence microscopy images were taken immediately after the compression of the substrate. (**f**) Colour map of the expected orientation angle of the active probes from the analytical solution of the first principal stress on the gliding assay substrate with a hole. (**g**,**h**) Colour maps of the orientation angle of (**g**) active probes and (**h**) non-motile probes; the angles were measured with respect to the stretch axis. From the colour maps, distribution of the compressive stress can be estimated. Scale bar, 500 μm.

We demonstrated the capability of the active probes for sensing surface mechanical deformation of soft materials by visualizing a stress field with a greater complexity than a uniform stress field (Fig. 6a); this stress field has been analytically solved^30^. We compared the stress field obtained from the analytical solution with that obtained from our experiments, and thus we investigated the validity of our method of sensing surface mechanical deformation of soft materials using active probes. A stress field with a hole (diameter: ∼1.0 mm) at the centre was visualized on a PDMS substrate (Fig. 6a); upon application of the inhomogeneous stress, the active probes would be oriented in accordance with distorted stress directions. The PDMS substrate with a hole was elongated (*ɛ*=75%, strain rate=0.6% s^−1^) and subsequently compressed to its initial length. After the application of the compressive stress, the active probes were oriented radially around the hole (Fig. 6a–e). The predicted stress direction calculated from theoretical principal direction (Fig. 6f) and the colour map^31^ which represents the distribution of the orientation angle of the active probes (Fig. 6g) revealed that the orientation direction of the active probes well corresponded with the theoretical one. We also performed the same experiment using non-motile probes, that is, in the absence of ATP. Upon deformation of the substrate the non-motile probes did not show any preferential orientation (Fig. 6h). Instead, the fragmentation and buckling of the probes were observed^18^,^19^, which suggests that dynamic condition is required to use microtubules as probes for sensing the mechanical deformation at the surface of a soft material (Supplementary Fig. 14). Thus, we successfully demonstrated the ability of the active probes for sensing surface mechanical deformation by visualizing the stress field of a substrate with a greater complex morphology.

## Discussion

By performing the *in vitro* gliding assay of microtubules on PDMS we demonstrated the use of gliding cytoskeletal filaments as active probes for sensing surface mechanical deformation of soft materials. Our work provides a useful and robust approach with high throughput, and spatiotemporal resolution for characterizing surface mechanical deformation of soft materials by using a large number of active microprobes and a simple microscope-based detection system. If some probes are detached from the surface of soft materials during investigation, other probes are able to detect and fill up the vacancy. Even if some of the active probes suffer from any defect, there are sufficient probes available to help characterize the surface mechanical deformation without compromising with the accuracy of measurement. Properties such as the length and rigidity of the probes are also easily tunable^32^,^33^ which in turn would widen the application of the probes. Compared with other methods such as speckle pattern measurement^34^ or photoelasticity measurement of materials^35^,^36^, our method allows direct visualization of direction of surface deformation of soft materials by separating it from internal deformation of the materials. Furthermore, since the active probes are always moving, after finishing one set of measurement, they are able to autonomously reset their state, which allows repeating the surface deformation measurement without any hysteresis from the previous measurements. This methodology is applicable not only for PDMS but also for other soft materials, which was confirmed by replacing the PDMS by polyurethane (PU), a material used to prepare artificial skin and heart valves^37^,^38^,^39^,^40^. Although PU was previously reported to be harmful for motor proteins at an ambient condition^41^, we successfully demonstrated the gliding assay of microtubules on the PU using the stretch chamber. We also confirmed that similar to the PDMS, gliding microtubules on the PU substrate can sense the deformation of the PU and preferentially align to an energetically preferable configuration (Supplementary Fig. 15). Despite the promises, some drawbacks of the proposed methodology are needed to be addressed in future. For example, the presented method is based on the *in vitro* gliding assay of cytoskeletal filaments and motor proteins which work in aqueous environment under limited conditions such as at a neutral pH, and within a narrow range of temperature. Denaturation of proteins due to prolonged employment even in these conditions may also hinder the application of our methodology. Moreover, our method requires a specialized chamber for ensuring inert atmosphere around the specimen along with a multistep preparation process as described in the experimental section, which is also unavoidable at present.

Nowadays, soft materials are gaining much attention in diverse applications for the chemical, biomedical and health care industries; thus, understanding the surface deformation is important to broaden the applications of soft materials^42^,^43^. By providing a new methodology for probing the mechanical deformation at the surface of soft materials, the present work should facilitate further elucidation of the surface science of soft materials. Furthermore, through stretching of the gliding assay substrate, dynamic control of the velocity and movement direction of microtubules was demonstrated without using any micropatterning method^44^,^45^,^46^,^47^. This system might be employed to simultaneously control the direction and velocity of gliding cytoskeletal filaments over a wide area to form large-scale groups^48^,^49^. As well as the importance in nanotechnology, use of the stretchable device allows one to have an *in situ* control of motor protein density, which provided us with a better understanding on the biophysical aspects of dynamic motor protein systems, for example, correlations among the directionality, smoothness and velocity of the gliding microtubules (see Supplementary Information for detail).

## Methods

### Preparation of tubulin and kinesin

Tubulin was purified from porcine brain using a high-molarity PIPES buffer (HMPB) (1 M PIPES, 20 mM EGTA, 10 mM MgCl_2_; pH adjusted to 6.8 using KOH). HMPB and BRB80 buffer were prepared using PIPES from Sigma, and the pH was adjusted using KOH^50^. GFP-fused kinesin-1 consisting of the first 560 amino acids of human kinesin-1 (GFP-kinesin) was prepared by partially modifying previously reported expression and purification methods^51^. Rhodamine-labelled tubulin was prepared using 5/6-carboxy-tetramethyl-rhodamine succinimidyl ester (TAMRA-SE; Invitrogen) according to the standard techniques^52^. Rhodamine-labelled tubulin was obtained by chemical crosslinking, and the labelling ratio was 1.0, as determined by measuring the absorbance of the protein at 280 nm and that of tetramethyl-rhodamine at 555 nm.

### Preparation of labelled microtubules

Rhodamine-labelled microtubules were obtained by polymerizing a mixture of rhodamine-tubulin (RT) and non-labelled tubulin (NT) (RT:NT=4:1; final tubulin concentration, 56 μM) at 37 °C for 30 min. The solution containing the microtubules was then diluted with motility buffer (80 mM PIPES, 1 mM EGTA, 2 mM MgCl_2_, 0.5 mg ml^−1^ casein, 1 mM DTT, 10 μM paclitaxel and ∼1% DMSO; pH 6.8).

### Set up of the stretch chamber

The stretch chamber equipped with a computer-controlled stepping motor (SGSP-13ACT-B0; Sigma-Koki) was developed to elongate or compress the substrate of the *in vitro* gliding assay of microtubules along the horizontal direction (Fig. 1a; Supplementary Fig. 1)^18^,^19^. An adjusting screw was installed on the top of the chamber to control the focus of the field of view during observation under a fluorescence microscope. To perform experiments keeping the specimen in the stretch chamber in an inert atmosphere the oxygen gas was removed away from the stretch chamber by passing humid nitrogen gas^20^. Flow rate of the gas was controllable by using two ball valves fixed at inlet and outlet of the chamber.

### *In vitro* gliding assay on PDMS

A sheet of PDMS substrate (6.8 × 5 × 0.05) mm^3^ (L × W × T) (Fuso Rubber Industry) was fixed to the movable stretcher of the stretch chamber (Supplementary Fig. 2a). The PDMS surface was plasma treated for 1 min by a plasma etcher (SEDE-GE; Meiwafosis) to make it hydrophilic. Anti-GFP antibody (Invitrogen) at 0.2 mg ml^−1^ (10 μl) was applied to the plasma-treated PDMS surface (Supplementary Fig. 2a). After incubation for 3 min, the PDMS surface was washed with 5 μl of casein solution (80 mM PIPES, 1 mM EGTA, 1 mM MgCl_2_, ∼0.5 mg ml^−1^ casein; pH adjusted to 6.8 using KOH) and incubated for 2 min. Then, 5 μl of GFP-kinesin solution of prescribed concentrations (∼80 mM PIPES, ∼1 mM EGTA, ∼1 mM MgCl_2_, ∼0.5 mg ml^−1^ casein, ∼1 mM DTT, ∼10 μM paclitaxel/DMSO, ∼1% DMSO; pH 6.8) was introduced and incubated for 3 min to allow binding of the kinesins to the antibodies. The PDMS surface was washed with 10 μl of motility buffer (80 mM PIPES, 1 mM EGTA, 1 mM MgCl_2_, 0.5 mg ml^−1^ casein, 1 mM DTT, 10 μM paclitaxel/DMSO, ∼1% DMSO; pH 6.8). Next, 10 μl of microtubule solution (at the tubulin concentration of 400 nM, ∼80 mM PIPES, ∼1 mM EGTA, ∼1 mM MgCl_2_, ∼0.5 mg ml^−1^ casein, ∼1 mM DTT, ∼10 μM paclitaxel/DMSO, ∼1% DMSO; pH 6.8) was introduced and incubated for 3 min, followed by washing with 20 μl of motility buffer. Then, 100 μl of ATP buffer (5 mM ATP, 80 mM PIPES, 1 mM EGTA, 1 mM MgCl_2_, 0.5 mg ml^−1^ casein, 1 mM DTT, 10 μM paclitaxel/DMSO, ∼1% DMSO; pH 6.8) was dropped on a cover glass (40 × 50 mm^2^; MATSUNAMI) fixed at the baseplate of the stretch chamber (Supplementary Fig. 2b). After closing the stretch chamber, humid nitrogen gas was kept passing through the chamber to remove oxygen from the chamber. After passing the nitrogen gas for 30 min, the stretcher part equipped with the PDMS substrate was moved downward until it touched the ATP buffer on the cover glass. The time of ATP addition was set as 0 min, and fluorescence microscopy observation was started. The aforementioned experiments were performed at room temperature.

### Elongation or compression of the gliding assay substrate

The stretch chamber was used to elongate or compress the substrate of *in vitro* gliding assay of microtubules (PDMS). After sample preparation the stretch chamber was mounted to the stage of an inverted fluorescence microscope. The PDMS substrate attached to the movable stretcher of the stretch chamber was elongated or compressed to the prescribed strain using the computer-controlled stepping motor at the prescribed strain rate. The gliding assay substrate was subjected to single step uni-axial deformation at different stretching strain (0, 10, 20, 30, 50, 100 and 130%) at a prescribed strain rate (0.6−30.0% s^−1^) after applying the ATP buffer.

### Microscopic image capture

The samples were illuminated with a 100 W mercury lamp and visualized with an epifluorescence microscope (Eclipse Ti; Nikon) using an oil coupled Plan Apo 60 × 1.40 objective (Nikon). Filter blocks with UV-cut specifications (TRITC: EX540/25, DM565, BA606/55; GFP-HQ: EX455–485, DM495, BA500–545; Nikon) were used in the optical path of the microscope to allow the visualization of samples while eliminating the ultraviolet portion of the radiation and minimizing the harmful effects of ultraviolet radiation on the samples. Images and movies were captured using a cooled CMOS camera (Neo sCMOS; Andor) connected to a PC. To monitor a field of view for more than several minutes, ND filters (ND4, 25% transmittance) were inserted into the illuminating light path of the fluorescence microscope to avoid photobleaching.

### Estimation of kinesin density by QCM measurement

Kinesin solutions of different concentrations were applied to the gold-coated crystals of a QCM, before which surface of QCM sensor was coated with anti-GFP antibody in each case. Using ‘Sauerbrey equation' density of kinesin deposited on the gold-coated QCM crystal was determined for each kinesin concentration. Next, we directly observed the kinesin-coated surface of QCM sensor by using fluorescence microscope and fluorescence intensity of kinesins was measured for all the kinesin concentrations. A standard curve was prepared from which we obtained a linear correlation factor between fluorescence intensity and kinesin density as: (kinesin density)/(fluorescence intensity)=8.47 molecule·μm^−2^·a.u.^−1^ (a.u. stands for arbitrary unit of fluorescence intensity). Using the standard curve (the linear correlation factor) and fluorescence intensity, kinesin density on PDMS substrate was determined at different conditions.

### Image analysis for gliding assay

Movies of the gliding assays of the microtubules captured under a fluorescence microscope were analysed using Adobe Photoshop CS6 and Image J, including ‘Image J plugin Orientation J' (http://bigwww.epfl.ch/demo/orientation/)^31^. Specifically, the Image J plugin Orientation J, which quantifies the anisotropy of microtubules by calculating the pixel intensity of a specified area of (2,560 × 2,160) pixels, was used to obtain colour map of the angles between microtubule and the stretch axis shown in Fig. 6g,h.

### Simulation method

The simulation method was adopted from a previous work^28^, and modified for this study. Briefly, we simulated the three-dimensional movement of microtubules propelled by kinesin motors on compression of the substrate. The active probes were subjected to the constraint:


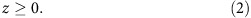


We consider the active probes to be infinitely thin and inextensible semi-flexible bead-rod polymers with a bending stiffness of 4.4 pN μ*m*^2^ (Supplementary Fig. 12). The length of active probes was set at 10 μm, corresponding to the most frequent value of the active probe length in our experiment, and each active probe consisted of 10 rigid segments (Supplementary Fig. 11a). Active probe movement was simulated with Brownian dynamics under the constraint of fixed segment length^53^.

Kinesin motors were randomly distributed over the allowed surfaces by specifying the positions of the kinesin tails. The surface density of kinesins was 40 μm^−2^. To ensure reaching steady state, active probes were allowed to move for 50 s corresponding to a distance equal to an active probe length before compression of substrate. The compression of the substrate was simulated by changing positions of kinesin tails (*x*_*i*_ (*t*), *y*_*i*_ (*t*)) with the following expressions (Supplementary Fig. 11b):


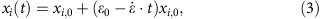



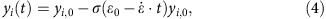


where *ɛ*_0_ is the initial strain, 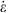 is the strain rate (5.0% s^−1^), *σ* is the Poisson ratio, *x*_*i*,0_ and *y*_*i*,0_ are the positions of kinesin tails without any strain. According to our measurement, the Poisson ratio was a function of strain (*ɛ*) and can be well described as:


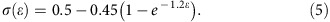


If an active probe segment came close to a kinesin motor tail within a capture radius (*w*=20 nm)^17^, the kinesin motor was assumed to be bound to the active probe segment, and the position of the motor head was specified on the active probe segment. The bound kinesin acted as a linear spring between the motor head and tail with the spring constant of 100 pN μm^−1^ and with zero equilibrium length, and exerted a pulling force on the active probe segment. The pulling force was divided into two forces which acted on the two beads located at either end of the active probe segment where the kinesin motor was bound, under the condition that the total force and torque on the segment remained the same. The head of the bound kinesin motor moved towards the active probe plus end (Supplementary Fig. 11a) with a force-dependent velocity expressed as


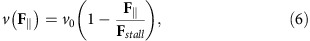


where *v*_0_ is the translational velocity without applied forces (Supplementary Fig. 11c), **F**_**||**_ is the component of the pulling force along the active probe, and **F**_stall_ is the stall force of kinesin motors. *v*_0_ was set at 0.2 μm s^−1^; **F**_stall_ was set at 5 pN for the kinesins^54^. A kinesin motor bound to an active probe was assumed to detach either when the pulling force exceeded the detachment force of 7 pN or when the kinesin reached the plus end of the active probe^55^. We neglected spontaneous dissociations of kinesin motors from active probes. The simulation result was visualized with ParaView (http://www.paraview.org/).

### Data availability

The data supporting the findings of this study are available from the corresponding author upon request.

## Additional information

**How to cite this article:** Inoue, D. *et al*. Sensing surface mechanical deformation using active probes driven by motor proteins. *Nat. Commun.* 7:12557 doi: 10.1038/ncomms12557 (2016).

## Supplementary Material

Supplementary Information Supplementary Figures 1-15, Supplementary Notes 1-2 and Supplementary References

Supplementary Movie 1 *In vitro* gliding assay of active probes on PDMS. Active probes (microtubules) were readily damaged by the reactive oxygen species (ROS) when the *in vitro* gliding assay was demonstrated using the conventional scavenger system (left). In contrast, employment of an inert atmosphere in the gliding assay successfully prevented the damage of the active probes by ROS and as a result the probes were motile for a prolonged period of time (right). Scale bar: 25 μm.

Supplementary Movie 2 Orientation of the active probes after elongation of the gliding assay substrate. The movie was acquired just after elongation of the substrate with stretching strain of 130%. Scale bar: 20 μm.

Supplementary Movie 3 Orientation of the active probes after compression of the gliding assay substrate. The movie was acquired just after compression of the substrate with compression strain of 130%. Scale bar: 20 μm.

Supplementary Movie 4 Change in the moving direction of a simulated active probe upon compression of the gliding assay substrate. The length of the simulated active probe is 10 μm and the applied strain is 130%.
