# Supplementary material for: Sensing surface mechanical deformation using active probes driven by motor proteins
Source: Nat Commun. 2016 Oct 3;7:12557. doi: 10.1038/ncomms12557 (PMC5059436; doi:10.1038/ncomms12557)
Supplement: Supplementary Information — Supplementary Figures 1-15, Supplementary Notes 1-2 and Supplementary References [file ncomms12557-s1.pdf]

Supplementary Figures

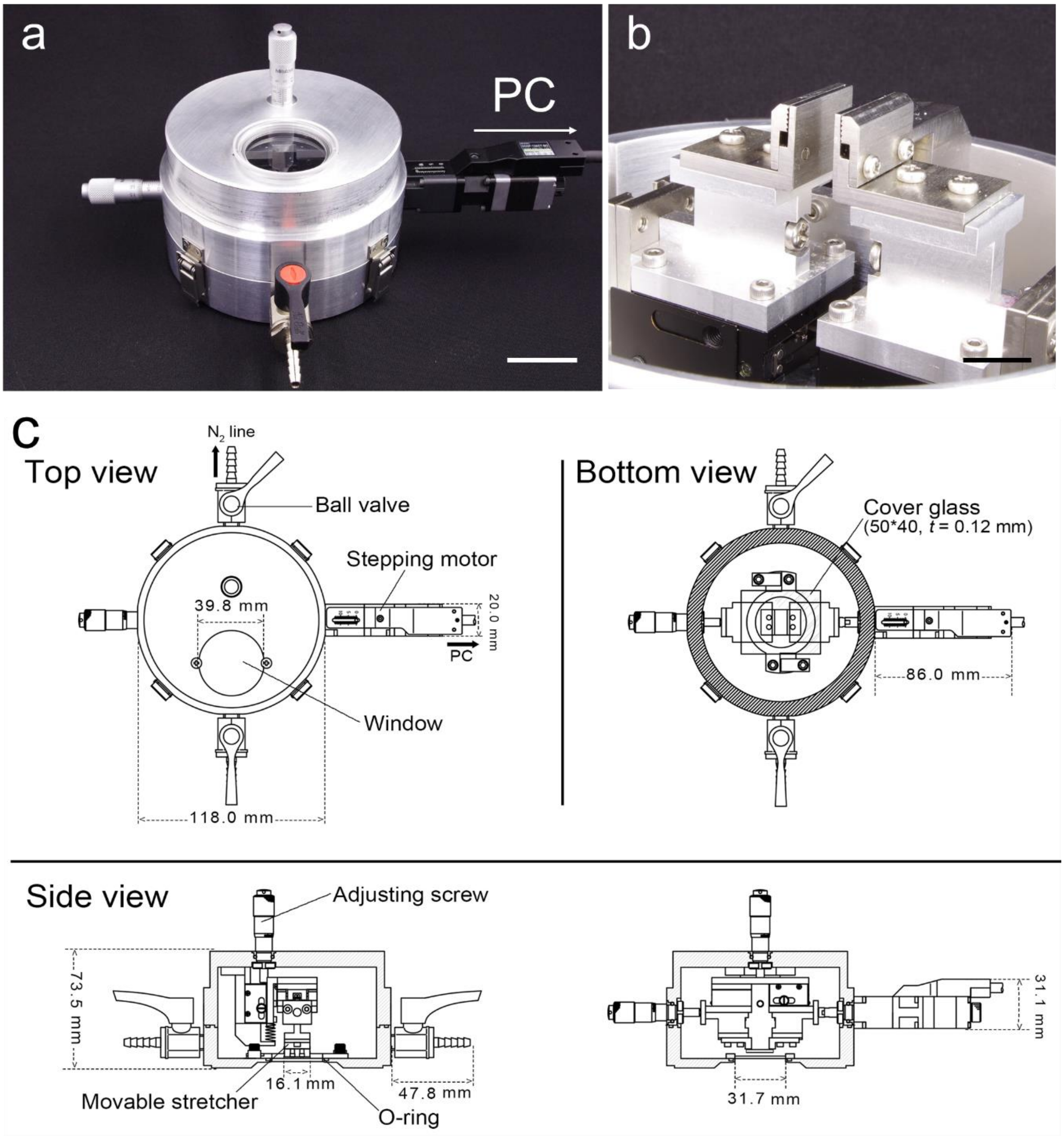

Supplementary Figure 1: Images and design of the stretch chamber used to elongate and compress the substrate of *in vitro* gliding assay. (**a, b**) outer and inner view of the stretch chamber. Scale bar: 30 mm (**a**), 10 mm (**b**). (**c**) Design of the stretch chamber. The chamber is equipped with one computer controlled stepping motor that was used to elongate or compress the substrate (PDMS) along the horizontal direction. Adjusting screw installed on the top of the chamber allows controlling the focus of the field of view during observation under a fluorescence microscope.

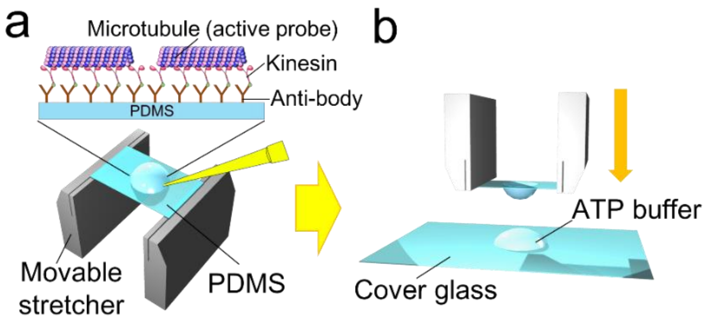

Supplementary Figure 2: Schematic diagrams showing the method of demonstration of the *in vitro* gliding assay on the PDMS substrate. (**a**) Method followed for applying samples to the surface of the PDMS substrate fixed to the movable stretcher of the stretch chamber. (**b**) Illustration showing how the ATP buffer was introduced to the gliding assay system in order to initiate motility of active probes on the kinesin coated PDMS substrate.

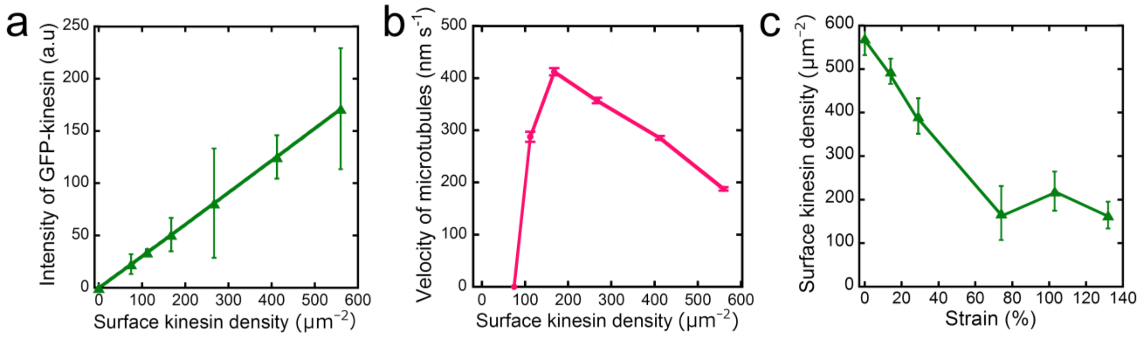

Supplementary Figure 3: Relationship between surface kinesin density and velocity of active probes on the PDMS substrate. (**a**) Standard curve shows the relationship between the density of GFP-kinesin on the PDMS substrate and fluorescence intensity. An area of (281.6×237.6) μm^2 was considered for measuring the fluorescence intensity. The density of GFP-kinesin was estimated by using Quartz Crystal Microbalance (QCM) as described in the methods. (**b**) Velocity of the active probes at different surface GFP-kinesin density. (**c**) Change in density of GFP-kinesin on the PDMS substrate on changing stretching strain at the substrate. Kinesin density was estimated from the standard curve shown in **a** by using the fluorescence intensity at PDMS substrate at different stretching strains. Error bar: standard deviation (**a, c**) and standard error (**b**).

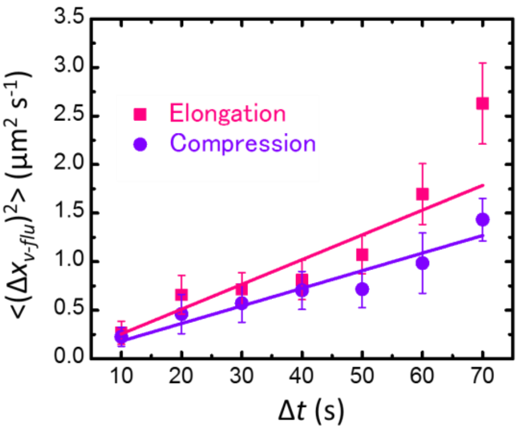

Supplementary Figure 4: Smoothness of movement of the active probes. Averaged mean-square deviation of the distance travelled by active probes as a function of the time lag after elongation and compression of the gliding assay substrate. The lines were drawn by least-square method. Error bar: standard error.

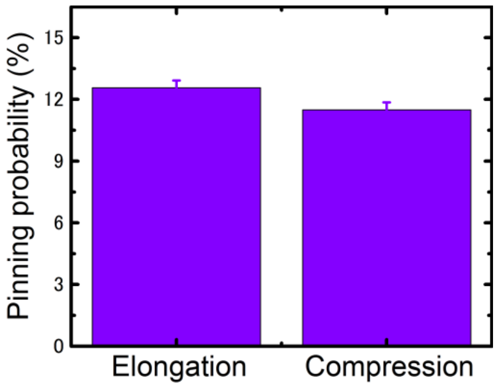

Supplementary Figure 5: Pinning probability of active probes after elongation and compression of the gliding assay substrate. To measure the probability, we acquired a movie of a single active probe movement and divided the number of movie frames, which showed pausing of the active probe, by total number of movie frames. Error bar: standard error.

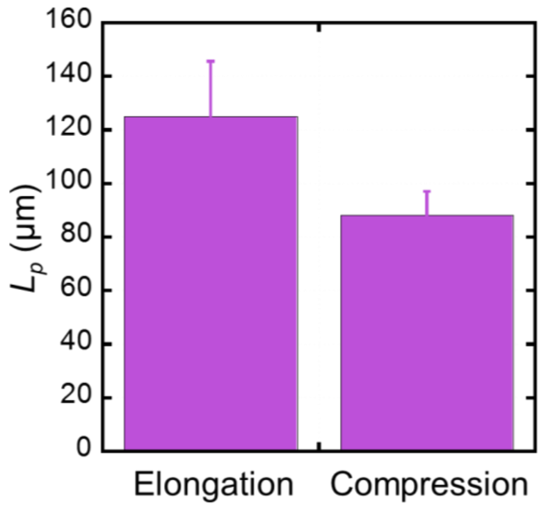

Supplementary Figure 6: Comparison of persistence length, L_p of the active probes after elongation and compression of gliding assay substrate. The substrate was elongated at a stretching strain of 130%, and compressed back at a rate of 5.0% s^{-1}. The L_p of the active probes after elongation of the gliding assay substrate was significantly higher than that found after compression of the substrate ($p<0.05$). Error bar: standard error.

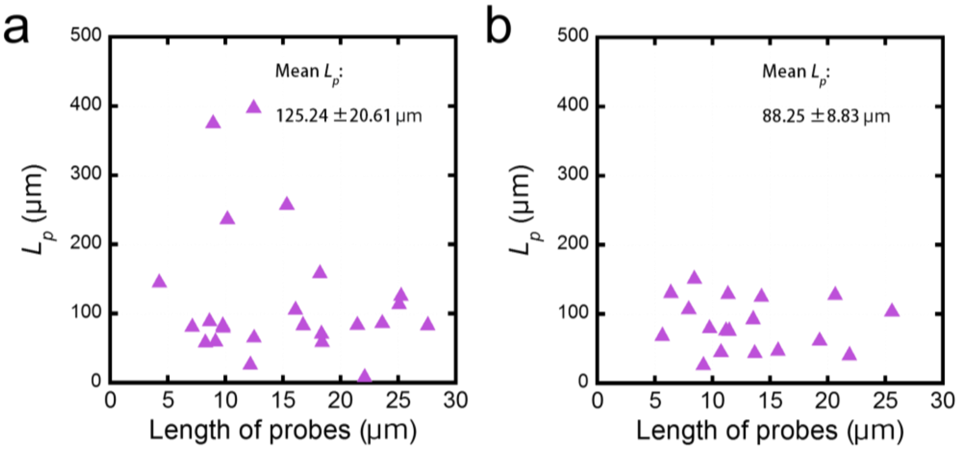

Supplementary Figure 7: Relationship between length and persistence length, L_p of active probes. The lengths of active probes were 13.9 ± 6.6 μm (average ± standard deviation) and 13.1 ± 5.6 μm after elongation and compression of the substrate respectively. The L_p of the probes with different lengths were measured after (**a**) elongation and (**b**) compression of the gliding assay substrate at a stretching statin of 130%.

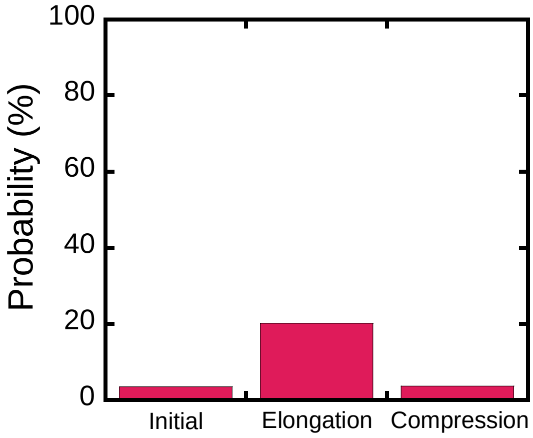

Supplementary Figure 8: Probability of fluctuation or detachment of active probes. The probability of fluctuation or detachment of active probes was higher after elongation than before elongation (initial) and after compression of the substrate at a stretching strain of 130%. The probability of fluctuation was measured by dividing the number of fluctuating and detaching active probes by the total number of active probes in an area of (77.0 × 77.0) μm^2.

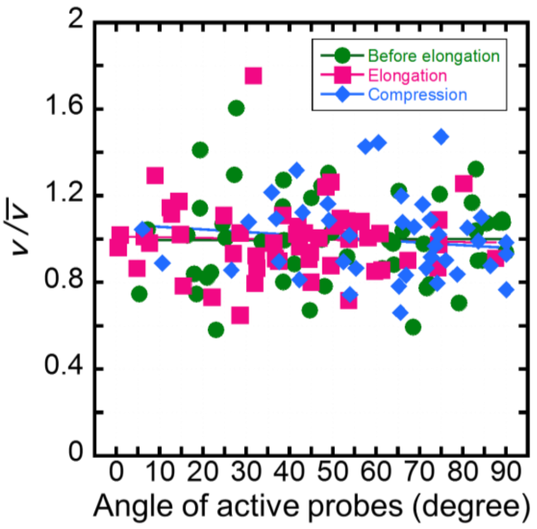

Supplementary Figure 9: Distribution of the normalized velocity, $v/\bar{v}$ at different moving directions of the active probes before elongation, after elongation and compression of the substrate. Velocity of the active probes, v was normalized by the average velocity, $\bar{v}$ before elongation, after elongation and compression of the substrate in respective case. In spite of a little heterogeneity in kinesin distribution there was no significant difference in the distribution of velocity of the active probes before and after elongation of the PDMS substrate ($p>0.05$).

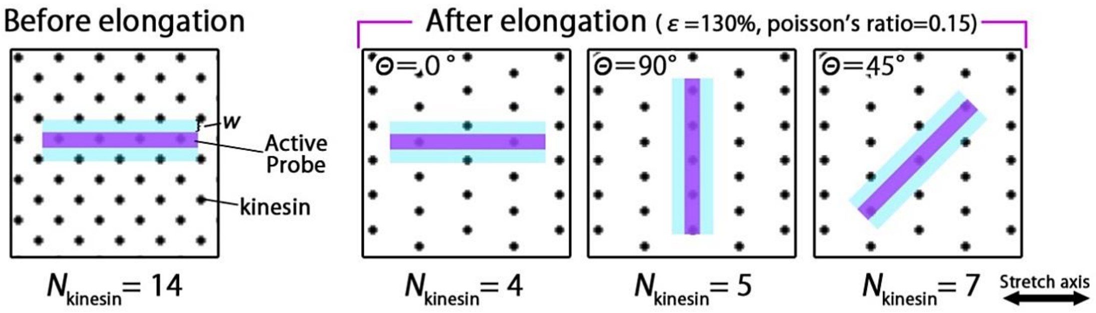

Supplementary Figure 10: Schematic diagrams showing the probable number of kinesin bound to an active probe against their moving direction under the heterogeneity of surface kinesin density after elongation of the substrate. The purple bar represents an active probe with the dimension of (260 × 25) nm^2. Kinesins are represented by the black dots. Initial kinesin density is set at 560 molecule μm^{-2}. Note that in the figure, only a limited part is shown. Kinesins are attached to the active probe in the range of radius w =20 nm from its position which is shown by the blue area around the active probe. After elongation of the substrate at a stretching strain of 130%, total number of kinesins attached to the active probe decreased. Considering the Poisson's ratio of the PDMS substrate, there is a little difference in the number of kinesin attached to the active probe between its perpendicular (θ = 90°) and parallel orientation (θ = 0°).

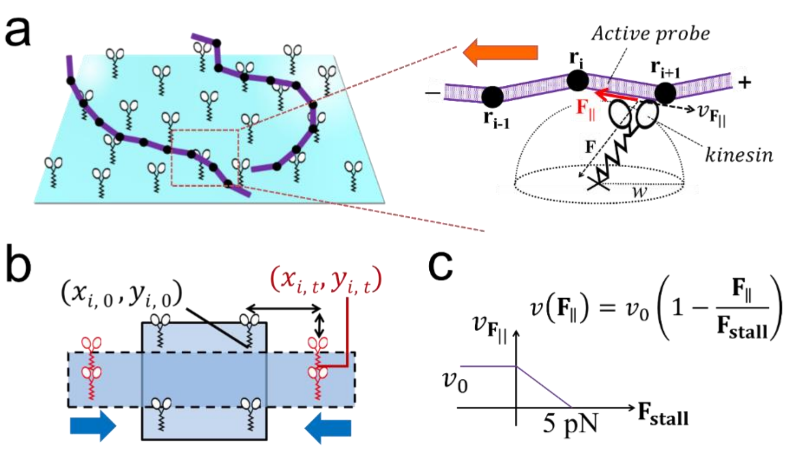

Supplementary Figure 11: Schematic diagrams of simulated active probes moving on a kinesin coated substrate. (**a**) Length of active probes was set at 10 μm which consisted of 10 rigid segments. Note that only a small section of an active probe is shown in the right figure because it is very large compared to the kinesins. The fixed tail of the *i*-th kinesin binds to an active probe in the range of a radius, *w* =20 nm from the ‘X’ mark. (**b**) Schematic diagram showing compression of substrate in the simulation. (**c**) Kinesin is a plus end directed motor moving along active probes with the velocity of $v(\mathbf{F}_{||})$. $\mathbf{F}_{||}$ indicates the component of the pulling force of kinesin, **F** along the active probe.

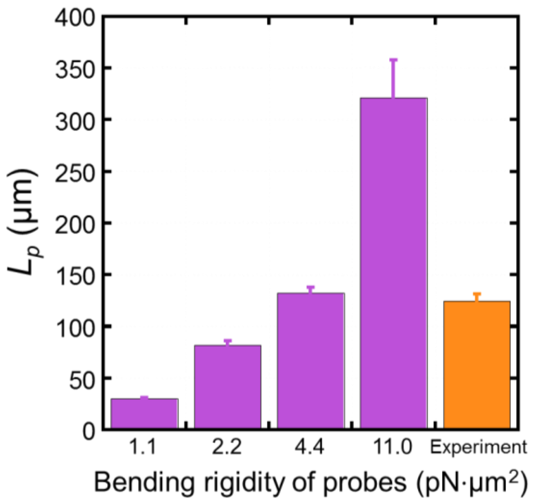

Supplementary Figure 12: Simulated path persistence length as a function of bending rigidity of probes. Since reported values of the bending rigidity of microtubules (probes) vary over a few orders of magnitude[1], we tuned the value of the bending rigidity to get a value of path persistence length consistent to that measured in our experiment. We simulated movements of probes having various values of the bending rigidity without the contraction of the substrate, and measured path persistence length of the probes (purple). We found that the bending rigidity of 4.4 pN·µm^2 gives a path persistence length consistent to that experimentally measured (orange). Hence, we used the bending rigidity of 4.4 pN·µm^2 for the simulation of probe movements on a contractile substrate. Error bar: standard error.

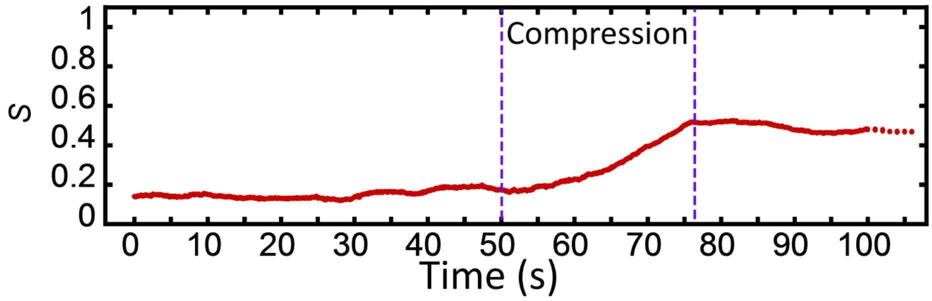

Supplementary Figure 13: Time course of the *S* of a simulated active probe under stretching and compression of the gliding assay substrate. The substrate was initially elongated at a stretching strain of 130%, and it was then compressed back to the initial length between 50 and 76 s at a strain rate of 5.0% s^{-1}.

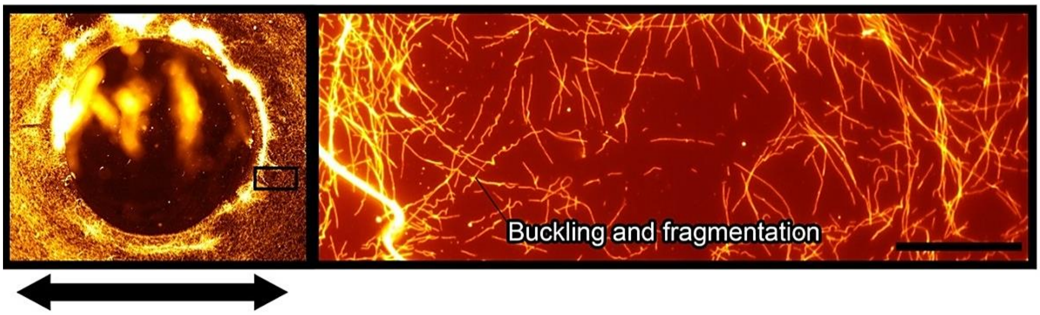

Supplementary Figure 14: Orientation of non-motile probes in an inhomogeneous stress field. Fluorescence microscopy images show buckling and fragmentation of the non-motile probes (in the absence of ATP) when the PDMS substrate with a hole was deformed. Scale bar: 100 μm.

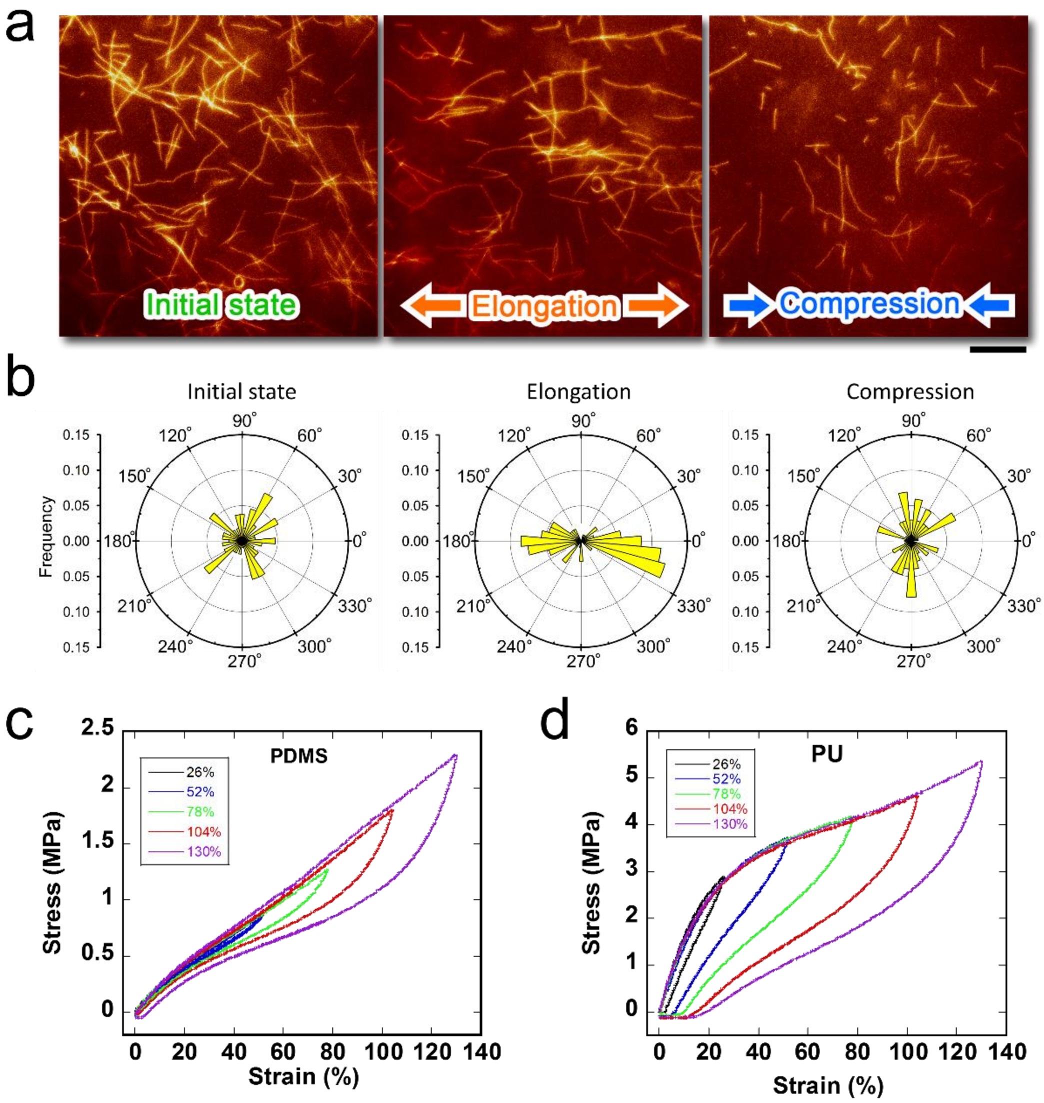

Supplementary Figure 15: Change of direction of movement and orientation of the active probes, gliding on polyurethane (PU), upon elongation and compression of the substrate. (**a**) Fluorescence microscopy images showing orientation of active probes at different substrate conditions. The active probes moved randomly on the PU substrate before the substrate deformation (left). The active probes were oriented parallel (middle) and perpendicular (right) to the direction of substrate elongation and compression respectively. For both the elongation and compression, 130% strain was applied to the substrate at the strain rate of 0.6% s^{-1}. Scale bar: 10 μm. (**b**) Circular histograms of orientation angle distribution of the active probes corresponding to the fluorescence microscopy images shown in **a**. (**c, d**) Tensile loading and unloading curve of PDMS and PU used in this work for different strains, as indicated in the insets, at a strain rate of 0.6% s^{-1}. A large hysteresis observed for the PU, which is absent for PDMS, can account for the scattered distribution of orientation angles of active probes upon compression of the PU substrate.

Supplementary Note

Supplementary Note 1: Importance of inert atmosphere in demonstrating deformation of the gliding assay substrate

By performing the *in vitro* gliding assay of microtubules on a soft and deformable substrate polydimethylsiloxane (PDMS) we validated our concept that motor protein kinesin driven microtubules can be used as active probes for characterizing surface mechanical deformation of soft materials. Here we have used a stretch chamber which we developed based on previously reported inert chamber system[2]. By employing an oxygen and reactive oxygen species (ROS) free inert atmosphere, we have performed the *in vitro* gliding assay of microtubules on the PDMS substrate and monitored the microtubules under a fluorescence microscope for a prolonged period of time (Supplementary Movie 1). The inert atmosphere is useful for performing gliding assay of microtubules not only on the PDMS substrate but also on other elastomeric materials, such as polyurethane (PU). Supplementary Fig. 1 shows the images and design of the stretch chamber that we used in order to mechanically deform the substrate of the *in vitro* gliding assay of microtubules. Similar to the case of inert chamber system, specimens are kept in oxygen and ROS free atmosphere while performing the *in vitro* gliding assay on a deformable substrate using the stretch chamber. The substrate e.g., PDMS was fixed to the movable stretcher of the stretch chamber (Supplementary Fig. 2) and upon application of tensile and compressive strain at the PDMS substrate the moving direction and velocity of the gliding microtubules changed. Deformation of the gliding assay substrate changed the kinesin density on the substrate which consequently altered the velocity of the active probes (microtubules) as shown in the Supplementary Fig. 3.

Supplementary Note 2: Dynamic behaviour of microtubules under mechanical stress

In this work, we characterized surface mechanical deformation of a soft material by employing microtubules as active probes which were gliding on the kinesin coated surface of the soft material. This work also offers a unique opportunity to investigate the dynamic behaviour of microtubules under mechanical perturbation which is of biophysical importance. Therefore, based on our investigations, here we correlate the directionality, smoothness of movement and velocity of the active probes to the stretching or compression of the substrate (Supplementary Fig. 4-10). With the help of simulation studies on the *in vitro* gliding assay of active probes, we have clarified the mechanism of sensing surface mechanical deformation of

the gliding assay substrate by the active probes (Supplementary Movie 4 and Supplementary Fig. 11-13). Self-propelling ability of the probes is found a pre-requisite for characterizing the surface mechanical deformation of soft materials (Supplementary Fig. 14).

As discussed in the main text, the active probes are aligned to an energetically preferable orientation upon application of mechanical stress to the substrate of gliding assay. The aligned probes start to attain random configuration once the application of mechanical stress is stopped. We monitored the relaxation process and measured the relaxation time as discussed in the main text. A difference in the relaxation time, τ for elongation and compression of the substrate is observed which might be associated with the difference in kinesin density dependent velocity change of the active probes upon elongation and compression of the substrate. Here we consider mobility of the active probes and measure the straightness of movement and the fluctuation or detachment probability of the active probes with and without deformation of the substrate. From the trajectory of the active probes we determined the persistence length of the active probes, L_p which is a characteristic length that relates the bending rigidity of probes to the thermal energy around them and can be expressed by the following equation[3]-

$$L_p = \frac{EI}{kT} \quad \text{(Supplementary equation 1)}$$

Here, E and I are Young's modulus and second moment of inertia of a probe respectively; k is the Boltzmann constant and T is temperature. 'EI' is called the flexural rigidity and thus persistence length of the active probes can be considered a measure of the straightness of movement of the active probes. From our experiments, we obtained the L_p using the following equation[3]-

$$\mathrm{Cos}(\theta_{t+\Delta t} - \theta_t) = \exp(-\Delta t/t_p) \quad \text{(Supplementary equation 2)}$$

where $(\theta_{t+\Delta t} - \theta_t)$ represents the time course of the moving direction of active probes from t sec to the time after a time interval of Δt sec and t_p is the time which gives the L_p. The L_p was calculated by multiplying the t_p with the time average velocity of active probes. The L_p of the active probes was smaller after compression of the substrate compared to that after elongation of the substrate (Supplementary Fig. 6), which could be attributed to different kinesin density on the substrate after elongation and compression. Previously, it was reported that the rigidity of the probes is related to interaction with kinesins[4,5] and at a higher kinesin density condition rigidity of the probes was reported to decrease significantly. Since on compression of the substrate, the kinesin density on the substrate increases, this might has decreased the rigidity of the active probes and as a result the straightness of movement of the active probes also

decreased. However, unlike the straightness of movement, the τ increased after compression of the substrate compared to that after elongation of the substrate. Therefore, we also consider the effect length of probes on the lifetime of the nematic order parameter, S. We investigated the relation of the length of the probes to the L_p of the probes. The lengths of the active probes were 13.9 ± 6.6 μm (average ± standard deviation) and 13.1 ± 5.6 μm after elongation and compression of the substrate respectively. We calculated the L_p for the probes with different length after elongation and compression of the PDMS substrate, but we found no significant dependence of L_p on the probe length (Supplementary Fig. 7). Thus, it is plausible to conclude that the τ was more influenced by the velocity and the fluctuation or detachment of the probes than the length or mechanical property, e.g. rigidity of the probes in the presented conditions. In fact, we observed that more active probes showed fluctuation at their ends and detached from the surface on elongation of the substrate, probably due to the decreased kinesin density and some probes were also reattaching to the substrate (Supplementary Fig. 8). At the strain of 130%, the kinesin density on the substrate was approximately 200 μm^{-2}, and at this strain ~20% of the active probes were detached from the substrate; a similar tendency was previously reported in the literature[6]. Thus, the velocity and fluctuation of the active probes seem to be involved with the relaxation time and could be considered the reasons behind the observed decrease in the τ after elongation compared to that after compression of the substrate.

We also investigated the fluctuations of the movement of active probes after elongation and compression of the gliding assay substrate by calculating the mean-square deviation of the sliding displacement from the average[7]. The mean-square displacement deviation from the average as a function of time allows measuring the motional diffusion coefficient, D_{v-flu}which is a measure of the fluctuation of movement of the active probes[7,8] and is expressed by the following equation-

$$< \left(\Delta x_{v-flu}\right)^2 > = 2D_{v-flu} \cdot t \qquad \text{(Supplementary equation 3)}$$

where, x_{v-flu} is the velocity fluctuation of each active probe and t is time. Supplementary Fig. 4 shows the averaged mean-square deviation of the distance traveled by active probes as a function of time after elongation and compression of substrate. We found that the mean square displacement from the average distance travelled increases linearly with time both after elongation and compression of the substrate. The D_{v-flu} of the active probes after elongation and compression are found to be $1.46 \times 10^{-2} \pm 0.13$ and $0.90 \times 10^{-2} \pm 0.05$ $\mu m^2\ s^{-1}$ respectively, where no substantial difference in pinning probability was detected

(Supplementary Fig. 5). The D_{v-flu} of the active probes after elongation of the substrate confirms more fluctuation of the movement of active probes compared to that after compression.

In the main text we discussed the correlation between surface kinesin density and the velocity of the active probes which were changed in response to the applied stretching strain at the gliding assay substrate. Here, we discuss the contribution of the heterogeneity of kinesin density to the orientation of the active probes. When the substrate is elongated along the x-axis, one may suspect that it can cause heterogeneity of the surface kinesin density; normally, the kinesin density is expected to decrease along the x-axis and should increase along the y-axis. Thereby, the heterogeneity of the velocity of active probes along the axes may also arise due to the different kinesin density. To elucidate the contribution of the heterogeneity of velocity of active probes to their orientation, we compared the velocity of the active probes against different direction before and after elongation or compression of the substrate. To neglect the aforementioned change in the mean velocity with and without elongation of the substrate, we used normalized velocity, $v/\bar{v}$, where v is the velocity of each active probe moving toward different direction against stretch axis and $\bar{v}$ is the mean velocity of the active probes. However, contrary to our suspicion, there was no significant difference in the velocity distribution before and after elongation or compression of the substrate ($p>0.05$) (Supplementary Fig. 9). To explain the independence of the velocity of active probes to the heterogeneity of surface kinesin density, we illustrated a model showing the number of kinesin bound to an active probe at different conditions (Supplementary Fig. 10). According to the model, before elongation of the substrate, approximately 14 kinesins were attached to an active probe with a dimension of (260 × 25) nm^2 at the kinesin density of 560 μm^{-2}. Considering the Poisson's ratio of the PDMS substrate (=0.15 at the stretching strain of 130%), we evaluated the number of kinesin bound to the active probe for different moving directions. We found no significant difference in the number of kinesins bound to the active probes oriented perpendicular and parallel to the stretch axis. Therefore, the suspected heterogeneity of the kinesin density due to elongation of the substrate is negligible and does not contribute to the biased moving direction of the active probes, which in turn explains the disappearance of the orientation of the probes after the substrate deformation was stopped.

In this work, we established a system to apply mechanical stress at microtubules gliding on a kinesin coated elastomeric membrane. This system, at the same time, enables one to dynamically change density of motor proteins by deforming the scaffold. It has been reported

that in *C. elegans* embryo, cortical dynein regulates positioning of centrosome under polarized cortical flow of actomyosin which works as a dynamic scaffold for dynein[9]. The system developed in this work might be useful in studying the relation between microtubule alignment and force propagation at microtubules from a dynamic scaffold via motor protein by dynamically changing the scaffold and density of motor proteins, which in turn would help understand the detail mechanism of spatiotemporal positioning of centrosome and nucleus in cells.

Supplementary Reference:

1. Hawkins, M., Mirigian, T., Yasar, M. S. & Ross, J. L. Mechanics of microtubules. *J. Biomech.* **43**, 23–30 (2010).
2. Kabir, A. M. R., Inoue, D., Kakugo, A., Kamei, A. & Gong, J. P. Prolongation of the active lifetime of a biomolecular motor for in vitro motility assay by using an inert atmosphere. *Langmuir* **27,** 13659–13668 (2011).
3. Howard, J., *Mechanics of Motor Proteins and the Cytoskeleton* (Sinauer Associates, Sunderland, MA, 2001).
4. Kabir, A. M. R., Inoue, D., Hamano, Y., Mayama, H., Sada, K. & Kakugo, A. Biomolecular motor modulates mechanical property of microtubule. *Biomacromolecules* **15,** 1797–1805 (2014).
5. Wada, S., Kabir, A. M. R, Kawamura, R., Ito, M., Inoue, D., Sada, K. & Kakugo, A. Controlling the bias of rotational motion of ring-shaped microtubule assembly. *Biomacromolecules* **16**, 374–378 (2015).
6. Howard, J., Hudspeth, A. J. & Vale, R. D. Movement of microtubules by single kinesin molecules. *Nature* **342**, 154–158 (1989).
7. Nitta, T., Hess, H. Dispersion in active transport by kinesin-powered molecular shuttles. *Nano Lett.* **5**, 1337–1342 (2005).
8. Imafuku, Y., Toyoshima, Y. Y. & Tawada, K. Fluctuation in the microtubule sliding movement driven by kinesin in vitro. *Biophys. J.* **70**, 878–886 (1996).
9. De Simone, A., Nédélec, F. & Gönczy, P. Dynein transmits polarized actomyosin cortical flows to promote centrosome separation. *Cell Rep.* **14**, 2250–2262 (2016).
